# A Lecture on the Treatment of Gout

**Published:** 1906-12-15

**Authors:** A. P. Luff

**Affiliations:** Physician to St. Mary's Hospital


### Hospital Clinics.

### A Lecture

### ON THE TREATMENT OF GOUT.

By A. P. LUFF, M.D., F.R.C.P.(Lond.), Physician to St. Mary's Hospital.

Gout is a disease due to faulty metabolism producing an auto-intoxication which coincides with the deposit of sodium biurate in the joints and tissues. Unmerited importance has been attached to uric acid as a factor in the causation of gout. It should be clearly recognised that uric acid contains no toxic properties worth mentioning, and is only a sign of the disease and not the essence of it, just as is sugar in the case of diabetes. The contention that a meat diet is poisonous to the human body is preposterous. If meat is the poison that certain enthusiasts insist on, the healthiest and most enlightened nations on the earth would long ago have ceased to exist. In this country the tendency of people is to eat too much and to masticate too little, not only meat, but all other solid foods. In treating gout one has to treat the paroxysm; the sub-acute condition; the affected joints; and irregular or abarticular gout.

#### Treatment of Acute Gout.

In treating acute gout, efforts should be directed to the arrest of abnormal intestinal fermentation and the removal of the intestinal bacteria by administering at night 4 grains of calomel or blue pill, followed by a saline aperient in the morning. If the mercurial is given at once, the saline aperient should be administered seven or eight hours afterwards. The patient should have no food whatever, nothing but water, for the first twenty-four hours. The bed-clothes should be prevented from pressing on the great toe by the use of a cradle. To relieve local pain some anodyne lotion should be applied, but on no account should the ice-bag or any depletives be used. A weak alkaline lotion is better than a strong one, which latter is apt to irritate and cause vesicular rashes. A useful lotion is:—

| | |
|---|---|
| Carbonate or bicarbonate of sodium | 3 drs. |
| Tincture of opium ... ... ... | 2 oz. |
| Liniment of belladonna... ... ... | 2 ,, |
| Distilled water to ... ... ... | 8 ,, |

A small quantity of this lotion is poured into a saucer with an equal quantity of boiling water and applied to the painful part on lint. No oiled silk should be put over this dressing.

*Internal Treatment.*—Colchicum is the most valuable drug and large initial doses of it should be given in combination with citrate of potassium, thus:—

| | |
|---|---|
| Colchicum wine ... ... ... ... | 40m |
| Citrate of potassium ... ... ... | 40 grs. |

to be given at once. Every four hours afterwards 15 to 20 minims of the wine with the same dose of citrate of potash should be given. Under this treatment the severity of the pain vanishes in a remarkable manner. After the first three or four days of this treatment the following pill should be used:—

| | |
|---|---|
| Colchicine ... ... ... | 1-70 to 1-50 gr. |
| Extract of nux vomica ... ... ... | $\frac{1}{4}$ ,, |
| Extract of hyoscyamus ... ... ... | $\frac{1}{2}$ ,, |
| Extract of gentian ... ... ... ... | 1 ,, |

Large doses of colchicine are apt to set up griping and purging, and are not necessary. In sub-acute gout $\frac{1}{60}$ gr. and in chronic gout $\frac{1}{70}$ gr. of colchicine will be sufficient. The bowels should at this stage be kept only moderately open, free purging not now being necessary. This effect may be accomplished by the following pill:—

| | |
|---|---|
| Leptandrin ... ... ... ... ... | 1 gr. |
| Iridin ... ... ... ... ... | 1 ,, |
| Extract of henbane ... ... ... | 1 ,, |
| Extract of colocynth ... ... ... | $1\frac{1}{2}$ ,, |

If the pain is too great to permit sleep, administration of morphine or opium should be avoided as being apt to diminish the amount of urine, to derange digestion, and to check the metabolism of the liver. It is better to rely on veronal 7 gr., or trional 10 gr., or extract of henbane $1\frac{1}{2}$ gr., as measures for producing sleep. Veronal does not require to be repeated the following night.

*Sub-acute and Chronic Forms.*—Guaiacum resin is extremely valuable in treating the chronic forms of gout and in preventing their recurrence. A six-months' course of guaiacum resin will diminish the severity and the frequency of gouty attacks, and in a great many cases will altogether prevent their return. It fails in the hands of some practitioners because they give it in the nauseous form of the ammoniated tincture. It should be given in the form of the powdered resin made up in cachets, and should never be ordered in a compressed form. Five grains of the resin to begin with should be given in this way three times a day, increasing by one grain each week up to 10 grains, upon which patients are ultimately kept for about six months. In regard to the potassium salts, the citrate produces no effervescence in the stomach and is not so depressing as the bicarbonate. It tends to diminish the acidity of the urine, to prevent the deposition of sodium bi-urate in the kidney tissue, and to increase the elimination of urates. It has been previously pointed out that if a mineral water is used as a solvent of gouty deposits it is better not to employ those containing the sodium salts; but in cases where the liver is sluggish, these salts are of use. The lithium salts are not so useful as those of potassium and sodium on account of their greater toxicity and depressant action on the heart. Patients are constantly seen by medical men suffering from cardiac depression and dilatation of the heart resulting from continued consumption of lithium in compressed form.

*The Joints.*— The limb should be elevated, damp compresses applied, and the hot douche used. The foot may be dipped into a bath of common salt or the joint sponged with the same. The alternate application of hot and cold water, one minute each, continued for twenty minutes, has a stimulating effect upon the circulation of the affected joint, increasing tissue change and favouring absorption. Massage of the joint should be resorted to immediately after the douche. The salt pack is also efficacious. Flannel is soaked in a saturated solution of common salt and wrapped round the affected joint, covered with oiled silk and kept on all night. This is followed in the morning by massage. The battery 10 milliampères—the negative pole over the affected joint, the positive being placed elsewhere—is used in the treatment of œdema and deposits in joints. This treatment should also be followed by a few minutes' massage to hasten absorption and to quicken circulation. Massage with the iodide of potassium and soap liniment may also be used.

### Treatment of Some Forms of Irregular Gout.

Dyspepsia of a gouty kind may be treated with the ordinary remedies, bismuth, bicarbonate of sodium, and bitters. Taka-diastase is useful in cases of gouty dyspepsia. It is used in tablets each containing 2½ grains. It very rapidly ensures the digestion of the carbohydrate elements and has the great advantage of not interfering with the development of the diastetic ferments of the pancreatic juice. It should be given immediately before a meal.

Another form of dyspepsia which occurs in connection with gout is hyperchlorhydria, which, however, is not always of gouty origin. It is indicated by gastric discomfort two hours after a meal and is relieved by the next meal. The discomfort may simply amount to distension and oppression, or it may be a severe pain of a burning character, referred to the epigastrium, or to the shoulder blades. The effect of this excessive secretion of hydrochloric acid is to cause too rapid digestion of the proteid elements of the food, the starchy constituents are retained and the secretion of gastric juice is kept up without having any constituent present in the stomach with which it can combine. The proteid diet should be diminished and alkalies administered to neutralise undue acidity. Sodium and potassium salts do not give permanent relief. Hopogan ($MgO_2$) peroxide of magnesium is a powerful antiseptic and tends to relieve the constipation nearly always associated with hyperchlorhydria. It is given in doses of from 15 to 30 grains, one hour after food—and repeated three times a day. It is best administered in a little milk; it is a tasteless powder and gives almost immediate relief and has a curative effect if continued for several days.

*The Diet in Gout.*—The chief source of gouty infection is the alimentary track, and the growth of bacteria varies with the amount of proteid consumed. This explains why excessive proteid foods and especially such forms as are liable to bacterial change, and also why certain wines and beers exercise so strong an influence in the production of gout. It is the *excessive amounts* of proteids that is harmful. The ideal diet is one which will supply the true needs of the body and maintain it at its highest degree of physical and mental activity. The gouty should avoid rich meat soups: oxtail, turtle, mock-turtle, kidney, mulligatawny, hare, giblet; salmon, mackerel, eels, lobster, crab, mussel, salted fish, smoked fish, preserved fish, tinned fish; duck, goose, pigeon, high game; meats cooked a second time; hare, venison, pork, lean ham, sweetbreads, liver, kidney, salted, corned or cured meats, pickled meats, preserved and potted meats, sausages; all articles of food pickled in vinegar, all highly seasoned dishes and rich sauces; tomatoes, beetroot, cucumber, rhubarb, mushrooms, truffles; rich pastry, rich sweets, new bread, cakes, nuts, dried fruits, ices, ice-cream.

The following gives an indication of the diet suitable for gouty subjects: *Morning.*—Half a pint of hot water flavoured with a slice of lemon peel should be slowly sipped immediately on rising. *Breakfast*—A selection may be made of the following articles of diet according to the taste of the patient: Porridge and milk, whiting, sole or plaice, fat bacon, eggs cooked in various ways, dry toast or "Zwieback bread" thinly buttered, and tea infused for three minutes and then strained from the leaves. Fat bacon is digestible when grilled, but less so when boiled. Eggs should not be taken hard boiled. *Lunch and dinner*—Soups suitable for the gouty are vegetable *purées* and soups made by boiling beef-bones or mutton bones with vegetables and subsequently removing the fat which separates on cooling. These soups should not be thickened with farinaceous substances. The varieties of fish most suitable to the gouty are whiting, sole, turbot, plaice, smelt, flounder, grey mullet, and fresh haddock.

The birds that are admissible as articles of diet are chicken, pheasant, turkey, and game (not high). Butcher's meat, mutton, lamb, and beef should be taken at only one meal in the day, and even then in moderate quantity. Two vegetables may be taken at both lunch and dinner. Any of the ordinary vegetables may be taken, except those previously mentioned as best avoided, but those that are most likely to prove beneficial to gouty subjects are spinach, brussels sprouts, French beans, winter cabbage, savoy cabbage, turnip-tops, turnips, and celery. Potatoes may also be taken in moderate quantities. Stewed fruits or baked apples or pears may be taken every day at one meal.

Green vegetables as salads may be taken, provided oily dressings are avoided. A simple savoury may, if desired, be taken at the end of dinner, or a small quantity of cheese, if well masticated and if free from fungus or mould. *Night*—Half a pint to a pint of hot water, flavoured with a slice of lemon peel, should be slowly sipped before retiring to bed.

Any fruit which from experience is known to agree with the individual may be taken by gouty subjects. Apples and oranges generally agree best. Uncooked fruit should never be taken at a meat meal and is best consumed fasting and fairly early in the day as between breakfast and lunch. It should always be thoroughly masticated. Strawberries are frequently avoided by the gouty owing to their producing in some subjects a certain amount of temporary irritation of the skin, but such irritation generally passes off in a short time. In a few subjects, strawberries produce eczema or some other rash, but such cases merely represent idiosyncrasy to the special fruit and necessarily such individuals, whether gouty or not, should not eat strawberries. Except in those cases in which there is an idiosyncrasy to their use, they constitute a good article of diet for the gouty on account of their delicious flavour, their antiscorbutic properties, and their richness in potassium salts. It is, however, very necessary that they should be ripe and fresh. They are soon prone to decomposition, and in such a state they aid in the development of those intestinal fermentations which are so inimical to the gouty.

